# Binary decisions of artificial intelligence to classify third molar development around the legal age thresholds of 14, 16 and 18 years

**DOI:** 10.1038/s41598-024-55497-5

**Published:** 2024-02-26

**Authors:** Ademir Franco, Jared Murray, Dennis Heng, Anna Lygate, Debora Moreira, Jaqueline Ferreira, Djessyca Miranda e Paulo, Carlos Palhares Machado, Juliano Bueno, Scheila Mânica, Lucas Porto, André Abade, Luiz Renato Paranhos

**Affiliations:** 1 Division of Forensic Dentistry, Faculdade São Leopoldo Mandic, Campinas, Brazil; 2 Department of Therapeutic Stomatology, Institute of Dentistry, Sechenov University, Moscow, Russia; 3 Centre of Forensic and Legal Medicine and Dentistry, University of Dundee, Dundee, UK; 4 Division of Oral Radiology, Faculdade São Leopoldo Mandic, Campinas, Brazil; 5 Dentistry Programme, School of Dentistry, Federal University of Uberlândia, Uberlândia, Brazil; 6 Department of Federal Police, National Institute of Criminalistics, Brasília, Brazil; 7 Computer Vision Solutions, Rumina S.A., Belo Horizonte, Minas Gerais Brazil; 8 Computer Science, Federal Institute of Science and Technology, Barra do Garças, Brazil; 9 Department of Preventive and Social Dentistry, Federal University of Uberlandia, Av. Pará–1720, Bairro Umuarama, Uberlândia, MG 38405-320 Brazil

**Keywords:** Artificial intelligence, Age estimation, Forensic dentistry, Radiology, Oral anatomy, Forensic dentistry

## Abstract

Third molar development is used for dental age estimation when all the other teeth are fully mature. In most medicolegal facilities, dental age estimation is an operator-dependent procedure. During the examination of unaccompanied and undocumented minors, this procedure may lead to binary decisions around age thresholds of legal interest, namely the ages of 14, 16 and 18 years. This study aimed to test the performance of artificial intelligence to classify individuals below and above the legal age thresholds of 14, 16 and 18 years using third molar development. The sample consisted of 11,640 panoramic radiographs (9680 used for training and 1960 used for validation) of males (n = 5400) and females (n = 6240) between 6 and 22.9 years. Computer-based image annotation was performed with V7 software (V7labs, London, UK). The region of interest was the mandibular left third molar (T38) outlined with a semi-automated contour. DenseNet121 was the Convolutional Neural Network (CNN) of choice and was used with Transfer Learning. After Receiver-operating characteristic curves, the area under the curve (AUC) was 0.87 and 0.86 to classify males and females below and above the age of 14, respectively. For the age threshold of 16, the AUC values were 0.88 (males) and 0.83 (females), while for the age of 18, AUC were 0.94 (males) and 0.83 (females). Specificity rates were always between 0.80 and 0.92. Artificial intelligence was able to classify male and females below and above the legal age thresholds of 14, 16 and 18 years with high accuracy.

## Introduction

Dental age estimation is used to support the Courts in cases that involve adoption, the investigation of sport players, criminal accountability, asylum seekers, and retirement^1–3^. Experts’ reports on dental age estimation must conclude based on what has been officially requested. Adoption cases, for instance, usually require a conclusion to the question “*what is the age of the child?*” The answer to this question is provided in form a continuous variable, namely the estimated age. Civil and criminal accountability, and alleged unaccompanied/undocumented asylum seekers, on the other hand, do not necessarily depend on the continuous value represented by the estimated age. Instead, these cases depend primarily on the binary answer to the question “*is this person an adult?*”^4^.

Dental development is a parameter of choice for age estimation at least up to the age of 21.5 years^5^, when the third molars normally reach apical closure. Most of the age thresholds of legal interest worldwide are included in the age interval between late childhood and early adulthood. According to the European Union Agency for Fundamental Rights, the age of sexual consent in most of the Member States range from 14 to 18 years^6^. When it comes to the age of legal majority, most of the U.S. States have established the threshold at the age of 18, similarly to European and South American countries^7^. Exceptions include the States of Alabama, Nebraska and Mississippi^8^, in which the legal age threshold increases to 19 and 21, respectively.

To estimate the age using third molars, medicolegal facilities need radiological equipment. On radiographs, forensic odontologists may have two main pathways for age estimation: (I) qualitative analyses of tooth developmental stages and their classification into ordinal data^9–11^, and (II) metric analyses of the teeth based on linear measurements and ratios between tooth parts^12^. Stages allocated to the teeth can be used in regressive formulae^13^ or can be converted into tabulated scores^14^ to lead to an estimated age. Differently, tooth ratios are compared to reference cut-offs to indicate if a person is below or above a certain age threshold of legal interest^15^. All these methods have the advantage of being non-invasive since they are radiologic^16^. The need for several and sequential operator-dependent decisions and procedures, however, represent an important bias that can increase subjectivity and inherent error rates of dental age estimation methods.

The use of Convolutional Neural Networks (CNNs) has contributed significantly to health sciences^17–19^, especially for image-based diagnosis^20–22^. In the last few years, artificial intelligence entered the dental age estimation arena as an alternative to promote automation in the field^23–25^. Because this is the start of a new era in dental age estimation methods, studies in the field have been fundamental to understand the performance of artificial intelligence on tasks, such as the automated classification of third molars^25^. More recently, CNNs were tested in a forensic environment to provide a binary answer about sex estimation from dentomaxillofacial radiographs^26^. A step further in the field would be challenging the binary decisions of artificial intelligence with the task of classifying people below or above age thresholds of legal interest. In other words, radiographic dental age estimation could benefit from a reduction of operator-dependent procedures.

Based on the exposed, the present study established a diagnostic accuracy test to investigate the performance of artificial intelligence to classify individuals below or above the age thresholds of legal interest of 14, 16 and 18 years based on the radiographic aspect of third molar development.

## Material and methods

This study was designed observational and cross-sectional. The sample used in this study was collected retrospectively from an existing image database in Central-Western Brazil. There was no exposure of patients to ionizing radiation for the purpose of the present study. All methods were carried out in accordance with relevant guidelines and regulations, such as the Declaration of Helsinki, 2013. The methodological protocols inherent to the observational cross-sectional study model were approved by the Committee of Ethics in Human Research of Faculdade São Leopoldo Mandic. Informed consent to use radiographs, and sex- and age-related data were obtained after the enrolment of each patient at the dental clinic. Because the study had a retrospective sample collection from an existing image database, additional permission to access and collect data was granted by a legal guardian of patients’ radiographic images.

The inclusion criteria consisted of panoramic radiographs of male and female Brazilian individuals (n = 11,640) between the ages of 6 and 22.9 years. The exclusion criteria consisted of panoramic radiographs without information about the patient’s sex, date of birth and date of image acquisition; visible bone lesions; missing mandibular left third molar; and poor image quality. The eligibility criteria excluded 1693 radiographs from the original database (n = 13,333). The images were imported to an Elitebook 15.6" FHD Laptop with i5 (Hewlett-Packard, Palo Alto, CA, USA) for analysis.

**Figure 1:**
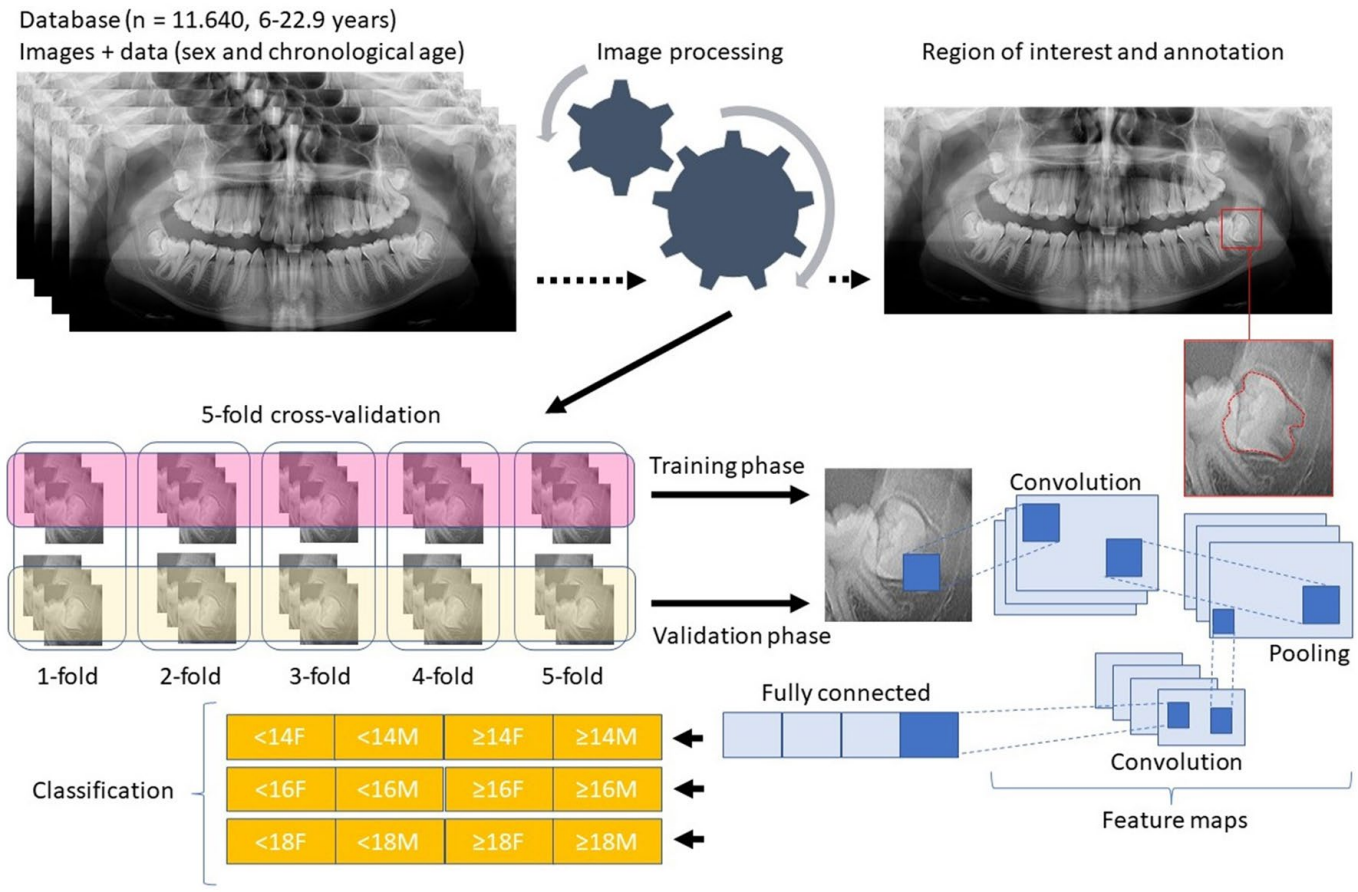
Workflow illustrating database sorting, image processing, image annotation (region of interest), cross-validation, training/validation phases, features maps and image classification based on age and sex.

Image analysis consisted, firstly, of annotations of the region of interest on each panoramic radiograph. To this end, Darwin V7 software package (Darwin V7 Labs, London, UK) was used^27^. The bounding-box tool within the software enabled the peripheral selection of the mandibular left third molar. With a semi-automated processing, the contour of the third molar moved from a box-shape to an anatomic outline of the selected tooth (Fig. 1). This procedure was repeated throughout the sample by four trained forensic odontologists^26^ supervised by a fifth one. The forensic odontologists were used to radiographic dental age estimation by means of panoramic radiographs both through metric and staging techniques. The reproducibility of the supervising observer, for instance, has been above 90% for metric analyses^1^ (for intra- and inter-observer reproducibility tests using the Intraclass Correlation Coefficient) and above 80% for staging techniques^9^ (for intra- and inter-observer reproducibility tests using the Weighted Kappa statistics) of third molars. The images were anonymized for annotation, hiding age and sex information. The software registered the annotations that were later tested for association with age.

The full set of panoramic radiographs was divided according to age thresholds of legal interest that are often considered in dental age estimation in the field of forensic odontology, namely the age of 14 years (to represent the age of sexual consent, for instance), the age of 16 years (to represent relative liability), and the age of 18 years (to represent the age of majority) (Table 1). Hence, initially, three single-problem scenarios were created: a binary classification of individuals that were < 14 or ≥ 14 years, < 16 or ≥ 16 years, and < 18 or ≥ 18 years. Because sex may play an important part in dental development, all the binary decisions were established for males and females, separately (Fig. 1).

**Table 1:** Distribution of the sample during the phases of training and validation based on age thresholds of legal interest.

Age threshold	Phase	n	n (male)	n (female)	Total
14 years	Training	2.880	1.440 720 < 14 years 720 ≥ 14 years	1.440 720 < 14 years 720 ≥ 14 years	3.600
	Validation	720	360 180 < 14 years 180 ≥ 14 years	360 180 < 14 years 180 ≥ 14 years	
16 years	Training	4.200	1.680 840 < 16 years 840 ≥ 16 years	1.680 840 < 16 years 840 ≥ 16 years	5.040
	Validation	840	420 210 < 16 years 210 ≥ 16 years	420 210 < 16 years 210 ≥ 16 years	
18 years	Training	2.600	1.300 650 < 18 years 650 ≥ 18 years	1.300 650 < 18 years 650 ≥ 18 years	3.000
	Validation	400	200 100 < 18 years 100 ≥ 18 years	200 100 < 18 years 100 ≥ 18 years	

Total training sample = 9.680; total validation sample = 1.960.

The radiographs used in this study were pre-processed preserving high-level of detail, spatial resolution, and quality. The CNN used in this study was DenseNet121. This network was selected because in a previous study^26^ it outperformed seven other CNNs for the purpose of forensic-radiographic image analysis (based on age and sex estimation in 100 epochs), namely InceptionV3, Xception, InceptionResNetV2, ResNet50, ResNet101, MobileNetV2, and VGG16. DenseNet121 is a well proven model, and is available from open sources (e.g. Pytorch, TensorFlow and Keras API). More specifically, DenseNet121 was employed with transfer learning. This is a variant of the learning process where the neural network weights are already pretrained on some other tasks and which are then adapted to the desired task. In this study, we used the transfer learning context adapting a pre-trained model (typically on a comprehensive dataset like ImageNet) to the specific domain of age assessment from radiographic images. This approach allowed the model to apply general knowledge, such as identifying basic features, to the new context (age estimation). In its implementation, DenseNet121 served as a fixed feature extractor, with its initial layers maintained for generic pattern recognition, while the upper layers are adapted to the specific task. Additionally, an alternative technique known as 'fine-tuning' adjusted more deeply the pre-trained model to the new dataset, potentially enhancing accuracy for the specific task^28,29^. Hence, the network borrows data or extracts knowledge from related fields to obtain the highest possible performance in the area of interest^30,31^.

To avoid overfitting and improve the generalizability of the evaluated model, due to the quantitative restriction of images in the data set, we used a computational framework (Keras)^32^ for pre-processing layers to create a pipeline of image data augmentation layers, which can be used as independent preprocessing code in non-Keras workflows^33^. These layers apply random augmentation transformations to a batch of images and are only active during training^33^. Table 2 presents each layer with its respective implemented parameters to avoid overfitting. A stochastic optimization algorithm (SGD) was applied to optimize the training process. We initially set a base learning rate of 1 × 10^−3^. The base learning rate was decreased to 6 × 10^−6^ with increased iterations. In the validation process, we used the k-fold cross-validation method^34,35^. In k-fold cross-validation, the dataset is divided into 'k' equal-sized subsets. For each round of validation, one subset is used for validation (testing), and the remaining 'k-1' subsets are used for training the model. This process is repeated 'k' times, with each subset used exactly once per validation. The final model performance is typically assessed by averaging the results from all 'k' folds. In this study, the dataset was divided into 5 (k) mutually exclusive subsets of the same size (five sets of 20% of the sample). This strategy creates a subset (20%) to be used for the tests and the remaining k − 1 (80%) is used to estimate the parameters (training). The five sets were dynamic, meaning that all the training samples had a different (randomly selected) dataset built from the original sample. Hence, images used during the training process were not used in the subsequent validation stage within the same k-fold training-test.

**Table 2:** Image data augmentation layers and parameters.

Layer	Parameter
Random Translation	Height_factor = 0.1, width_factor = 0.1, fill_mode = 'reflect'
Random Flip	Mode = 'horizontal_and_vertical'
Random Rotation	Factor = 0.1, fill_mode = 'reflect', interpolation = 'bilinear'
Random Contrast	Factor = 0.1

In order to quantify the performance of the CNN to classify individuals below or above the age thresholds of legal interest, we assessed the overall accuracy of the classification system, precision, recall and specificity. Precision is considered the agreement of true class labels with machine’s predictions. It is calculated by summing all true positives and false positives in the system, across all classes. Recall is the effectiveness of a classifier to identify class labels. It is calculated by summing all true positives and false negatives in the system, across all classes. Specificity is known as the true negative rate. This function calculates the proportion of actual negative cases that have gotten predicted as negative by our model. These metrics have been previously used to assess the performance of artificial intelligence models^26^. Additionally, we used Receiver Operating Characteristic (ROC) curves and Confusion Matrix approaches. A ROC curve is an illustrative outcome of the performance of a binary classification model. To accomplish a classification task, the model is set with different discrimination thresholds. In this study, the thresholds were the ages of 14, 16 and 18 years, individually considered and separately analyzed for females and males. The ROC curve considers and plots the true positive values (sensitivity) against false positive (1 − specificity) ones for each threshold. An overall quantitative representation of the plotted relationship is the Area Under the Curve (AUC). This is a number between 0 and 1, in which 0.5 means a random classification and 1 means a perfect performance of female and male classification below or above 14, 16 and 18, separately. To calculate the overall performance of each model, the average of each metric across all five folds was considered. For example, the average performance of DenseNet121 was the mean of the Loss, Accuracy, F1-Score, Precision, Recall, and Specificity values obtained from each of the five folds. This averaging method provides a more robust estimate of the model's performance, as it accounts for variability across different subsets of the data. It also helps in mitigating the overfitting issue and ensures that the performance metrics are not biased towards a specific part of the dataset. It must be noted that in the training stage, the weights of the model are updated during several iterations. We supervised each iteration and registered the weights with the best predictive power (determined by the overall accuracy metric). Computer processing was performed with a Linux machine, with Ubuntu 20.04, an Intel® Core(TM) i7-6800 K processor, 2 Nvidia® GTX Titan Xp 12 GB GPUs, and 64 GB of DDR4 RAM. All models were developed using TensorFlow API^36^ version 2.5 and Keras version 2.5 29. Python 3.8.10 was used for algorithm implementation and data wrangling^37^.

## Results

The accuracy rates to classify females and males below and above the age of 14 years were 0.863 and 0.872, respectively, while precision rates were 0.943 and 0.900, recall rates were 0.780 and 0.849, and specificity rates were 0.929 and 0.842, respectively. For the individuals classified around the age of 16 years, the accuracy rates were 0.828 and 0.883, precision rates were 0.850 and 0.917, recall was 0.831 for both females and males, and specificity rates were 0.802 and 0.896, respectively. When it comes to the age of 18 years, the metrics for females and males were: 0.829 and 0.939 (accuracy), 0.836 and 0.962 (precision), 0.860 and 0.901 (recall), and 0.768 and 0.927 (specificity), respectively (Table 3).

**Table 3:** Descriptive data of the performance metrics (accuracy, precision, recall and specificity) quantified in this study.

Age threshold	Sex	Accuracy	Precision	Recall	Specificity
14 years	Females	0.863	0.943	0.780	0.929
	Males	0.872	0.900	0.849	0.842
16 years	Females	0.828	0.850	0.831	0.802
	Males	0.883	0.917	0.831	0.896
18 years	Females	0.829	0.836	0.860	0.768
	Males	0.939	0.962	0.901	0.927

Total training sample = 9.680; total validation sample = 1.960.

**Figure 2:**
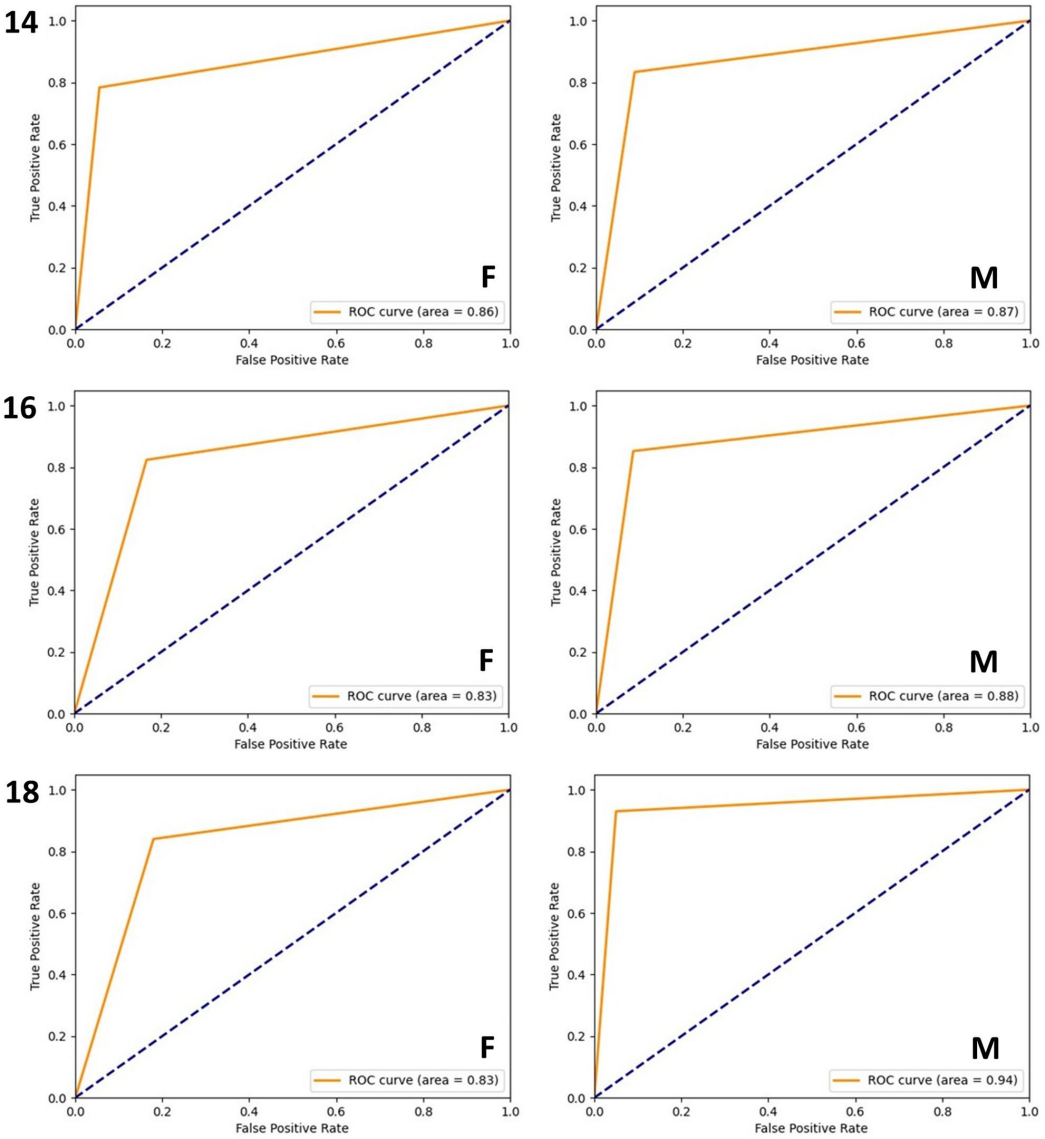
Receiver operating characteristic (ROC) curves for females (F) and males (M) classified below and above the ages of 14, 16 and 18 years.

The AUC for the females classified below and above the age of 14 years was 0.86, while for males it was 0.87. For the age threshold of 16 years, the AUCs for females and males were 0.83 and 0.88, respectively. For the classification of females below or above 18 years, the AUC was 0.83, while for males it was 0.94 (Fig. 2).

**Figure 3:**
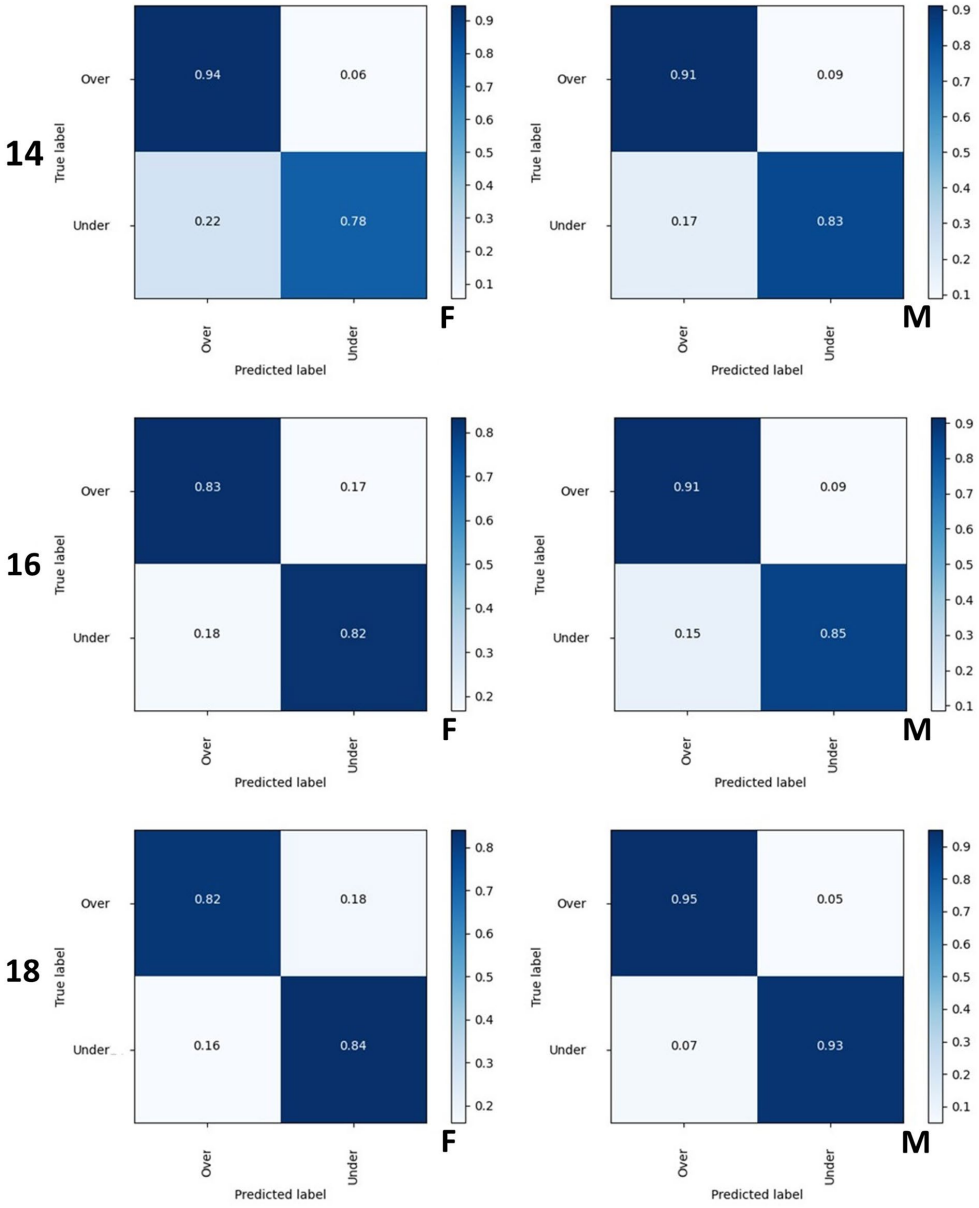
Confusion matrices obtained for females (F) and males (M) classified below and above the ages of 14, 16 and 18 years.

The outcomes of the Confusion Matrix for the correct classification of females below and above 14 years were 0.78 and 0.94, respectively. For males the correct classifications were 0.83 and 0.91, respectively. Around the age of 16 years, the values for the correct classification of females were 0.82 (below 16 years) and 0.83 (above 16 years). For males the values were 0.85 and 0.91, respectively. For the females, the correct classification of individuals below or above 18 years led to values of 0.84 and 0.82, respectively. Among males, the values reached 0.93 and 0.95, respectively (Fig. 3).

## Discussion

Operator-dependent procedures may lead to operator-dependent results^38^. Specific decisions in the field of forensic odontology rely on the experience of experts to visualize and interpret data, especially from radiographic images. The subjectivity behind these procedures may be more evident when dealing with the classification of patterns—and consequently when handling categorical/ordinal data. Dental staging is a common procedure in age estimation^39^. This non-metric approach to assess or estimate age from dental features can impact directly on people’s lives and human rights. In order to reduce the error potentially inherent to operator-dependent procedures, this study aimed to test dental age estimation performance transferring the staging decision-making from expert to artificial intelligence.

A recent study in the field of Cardiology^38^ have demonstrated that specific techniques performed to assess and measure hemodynamics depend on the experience of the operator. The authors highlighted that the studied procedure could impact on clinical decisions and treatment planning, for instance. Major focus on professional training, quality assurance, and repeatability is proposed^38^. The forensic field is not different, some of the staging techniques might be more difficult than others and require more advanced skills for image visualization and interpretation. An example of difficulty is to decide among a higher number of stages depending on the chosen dental age estimation method. The staging technique of Demirjian et al. proposed in 1973^40^, for example, is a well-known system based on eight sequential stages from A to H, in which the developmental stages for each tooth group (incisors, canines, premolar and molars) are described anatomically and with reference proportions between the formation of crown and root (e.g. stage F for uniradicular teeth indicates that “*the root length is equal or greater than the crown height*”). The system proposed by Moorrees et al.^41^, on the other hand, increases the number of stages by 43% naming the stages after the fractions of developed crown and root(s) (i.e. R¼, R½, R¾…). While the later system enables a more detailed description and categorization of the developmental process, it also increases the complexity of stage allocation because more options are available to the operator. To illustrate: Demirjian’s system has a single stage (H) dedicated to full apical closure, while Moorrees’ system has not only the stage for full apical closure (Ac) but also a preceding stage to indicate partial apical closure (A½). Deciding between these stages is a challenging task since the level of image detail required is not always available in extraoral radiographs.

To the present, the most recent study on the interface between artificial intelligence and dental age estimation was a systematic review^42^. The study has revealed that methods performed “manually” (operator only) tend to generate more marked overestimations (e.g. Demirjian’s method) and underestimations (e.g. Cameriere’s method), while fully automated systems based on deep learning have a more balanced performance. Our outcomes showed accuracy rates between 82 and 93% for all the three age categories (of legal interest). If we consider, for instance, a comparison focused in the age category of 18 years with the manual application of Cameriere’s I_3M_ method^12^, we will observe that previous studies with the same nationality (Brazilians) led to correct classification rates of 79.8%^43^, 80.2%^43^ and 87%^44^. Our accuracy for the classification of individuals below or above the age category of 18 years was 82% for females and 93% for males (AUC 0.83 and 0.94, respectively). These findings corroborate the optimal performance of artificial intelligence for age estimation not only because it reaches high accuracy, but also because it reduces labour and time in forensic tasks.

A scoping review published in 2021^45^ showed that applications of artificial intelligence in the field of forensic odontology are predominant in the subtopic of dental age estimation—compared to bite mark analysis, dental comparison (for human identification) and sex estimation. Despite being a subtopic of forensic odontology, dental age estimation is a broad field that includes methods for children, adolescents, and adults. The use of third molars as the sole indicators of age is mostly encouraged only when no other developing tooth can be used—usually between the ages of 16 and 21 years. This is to say that third molars have marked variations (in morphology and development timing) among people^46^ and are more difficult to visualize. These factors can influence dental age estimation performance. Boedi et al.^47^ listed some of the limitations of third molar staging through automated computer systems, such as the complex root morphology and the unwanted surrounding structures (e.g. adjacent bone and periodontal space) that are included in the region of interest during the process of digital image annotation. Some of these limitations were reduced in our study because the image annotation process was semi-automated and morphology-based, enabling the selection of third molar contour without including the mandible and mandibular canal, which happens when annotation is performed with a bounding box, for instance. Moreover, our study analyzed only third molars specifically to contribute to the challenging process of dental age estimation when dental development is scarce. By sampling young individuals, we were able to investigate the performance of artificial intelligence for three age thresholds of legal interest that are relevant not only for the Brazilian Law but in many countries abroad.

When it comes to the methodological settings used in our study, it is worth mentioning that when we evaluated the model trained using transfer learning, we observed that the vanishing gradient problem was mitigated, and the enhancement of feature propagation encouraged signal reuse, significantly reducing the number of parameters^48^. In DenseNet121, the classifier uses features of all complexity levels, which tends to give smoother decision boundaries. This also explains why DenseNet121 performs well when the training data is insufficient. Each layer in DenseNet121 receives all preceding layers as input, resulting in more diversified features and richer patterns. With a training process using a model pretrained on the ImageNet dataset, this model leverages features extracted by very early layers that are directly used by deeper layers throughout the same dense block. This functionality, combined with fine-tuning and the optimization of hyperparameters, has increased the predictive capacity of this architecture. Moreover, the customized implementations in the DenseNet121 architecture used in this study ensured network stability, even with a relatively small image dataset. This challenge was compounded by the distribution of samples across six classes according to legal age thresholds. It is important to note that the technique of data augmentation heavily relies on the balance of samples across the utilized classes for the recognition process. Therefore, considerable effort was made to ensure the best possible balance of sampling among classes to provide optimal performance.

Future studies in the field could propose the analysis of a larger dataset equally balanced per sex and age categories, as well as more tooth labels to test the performance of artificial intelligence in younger age groups. Other strategies could be designed to overcome the binary approach used in our study. From a technical perspective, it could be seen as a limitation of the present study, because the developed intelligence is not able to provide solutions as continuous variables—such as for the for question: “*what is the age of the examined person?*” Instead, the presented tool is trained to respond whether a person is below or above a specific age threshold of legal interest, namely 14, 16 and 18 years. From a practical perspective, the answer that is currently feasible is already enough to provide valuable contributions to the forensic practice. External validation in samples worldwide is encouraged.

## Conclusion

The artificial intelligence tested in the present study showed optimal performance to classify females and males below and above age thresholds of legal interest. The performance of this digital solution revealed higher accuracy rates than most dental age estimation methods based on operator decisions. The results were even better for males, which is a positive finding because this is the gender that is more frequently assessed in dental age estimation practice. Among the age thresholds of legal interest addressed in this study—14, 16 and 18 years—the best accuracy rates were observed around the age of legal majority in most countries (18 years), leading to a potential contribution to dental age estimation practice that involve asylum seekers and criminal/civil liability, for instance.

## Data Availability

The datasets used and/or analysed during the current study available from the corresponding author on reasonable request.
